# Temporal development of T cell receptor repertoires during childhood in health and disease

**DOI:** 10.1172/jci.insight.161885

**Published:** 2022-09-22

**Authors:** Angela M. Mitchell, Erin E. Baschal, Kristen A. McDaniel, Kimber M. Simmons, Laura Pyle, Kathleen Waugh, Andrea K. Steck, Liping Yu, Peter A. Gottlieb, Marian J. Rewers, Maki Nakayama, Aaron W. Michels

**Affiliations:** 1 Barbara Davis Center for Diabetes and; 2 Department of Pediatrics, University of Colorado School of Medicine, Aurora, Colorado, USA.; 3 Department of Biostatistics and Informatics, University of Colorado School of Public Health, Aurora, Colorado, USA.; 4 Department of Medicine and; 5 Department of Immunology, University of Colorado School of Medicine, Aurora, Colorado, USA.

**Keywords:** Autoimmunity, Immunology, Adaptive immunity, Diabetes, T cell receptor

## Abstract

T cell receptor (TCR) sequences are exceptionally diverse and can now be comprehensively measured with next-generation sequencing technologies. However, a thorough investigation of longitudinal TCR repertoires throughout childhood in health and during development of a common childhood disease, type 1 diabetes (T1D), has not been undertaken. Here, we deep sequenced the TCR-β chain repertoires from longitudinal peripheral blood DNA samples at 4 time points beginning early in life (median age of 1.4 years) from children who progressed to T1D (*n =* 29) and age/sex-matched islet autoantibody-negative controls (*n =* 25). From 53 million TCR-β sequences, we show that the repertoire is extraordinarily diverse early in life and narrows with age independently of disease. We demonstrate the ability to identify specific TCR sequences, including those known to recognize influenza A and, separately, those specific for insulin and its precursor, preproinsulin. Insulin-reactive TCR-β sequences were more common and frequent in number as the disease progressed in those who developed T1D compared with genetically at risk nondiabetic children, and this was not the case for influenza-reactive sequences. As an independent validation, we sequenced and analyzed TCR-β repertoires from a cohort of new-onset T1D patients (*n =* 143), identifying the same preproinsulin-reactive TCRs. These results demonstrate an enrichment of preproinsulin-reactive TCR sequences during the progression to T1D, highlighting the importance of using disease-relevant TCR sequences as powerful biomarkers in autoimmune disorders.

## Graphical abstract

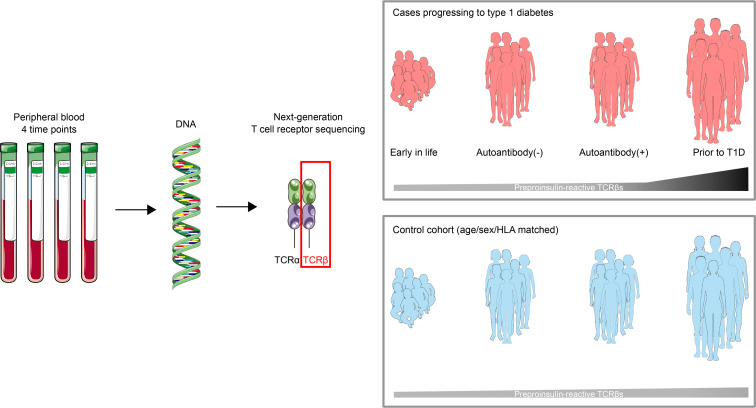

## Introduction

The T cell receptor (TCR) on the cell surface of T lymphocytes plays a central role in immune responses by the recognition of peptides in the context of major histocompatibility complexes (1, 2). Rearrangement of TCR α and β genes results in a vast and diverse repertoire of T cells within a given individual, allowing for recognition of millions of unique antigens. Advances in high-throughput sequencing technologies now enable deep sequencing of TCR-β chains from genomic DNA (3–5). Previous studies have analyzed TCR repertoires throughout the lifespan in a cross-sectional manner (6, 7), but none have longitudinally assessed the temporal changes in the repertoire beginning early in life and throughout childhood. The development of the TCR repertoire during childhood is beneficial for responding to foreign pathogens; however, recognition of self-peptides by T cells during this time is thought to contribute to development of autoimmune diseases such as type 1 diabetes (T1D), the immune-mediated form of diabetes.

T1D is a prototypical organ-specific autoimmune disease that develops in distinct stages marked by the presence of autoantibodies in peripheral blood targeting insulin and other pancreatic islet proteins such as glutamic acid decarboxylase (GADA), tyrosine phosphatase–related islet antigen-2 (IA-2A), and zinc transporter 8 (ZnT8A) (8, 9). Following islet autoantibody seroconversion, there is a period of time — generally years — prior to the development of hyperglycemia and clinical symptoms, and this requires lifelong treatment with exogenous insulin administration (10). Additionally, CD4 and CD8 T cells contribute to disease pathogenesis, as both cell types are present in the immune infiltrates of inflamed pancreatic islets from T1D organ donors (11–14). The disease course provides defined periods to measure immune responses prior to the development of islet autoantibodies, after seroconversion, and then at clinical T1D onset.

In this study, we longitudinally deep sequenced the TCR-β chain repertoire from peripheral blood beginning early in life from children genetically at risk for T1D and throughout childhood into adolescence. Cases developed islet autoantibodies and then went on to clinical disease, while age- and sex-matched nondiabetic controls did not develop autoantibodies or T1D. We show that the TCR-β chain repertoire is extraordinarily diverse early in life and contracts over time. From our large TCR-β data set, we then evaluated the hypothesis that a subset of self-antigen–specific TCR-β sequences obtained from the target tissue, inflamed pancreatic islets of human T1D organ donors, would be present in blood and accumulate throughout the stages of diabetes development. We show that there is a specific enrichment of TCR-β sequences known to respond to insulin and its precursor, preproinsulin (PPI), during the progression to clinical diabetes. The ability to sequence TCRs from peripheral blood DNA has significant relevance for monitoring disease progression in T1D and other autoimmune disorders with distinct stages.

## Results

### Diversity of the TCR-β chain repertoire decreases throughout childhood.

**Figure 1:**
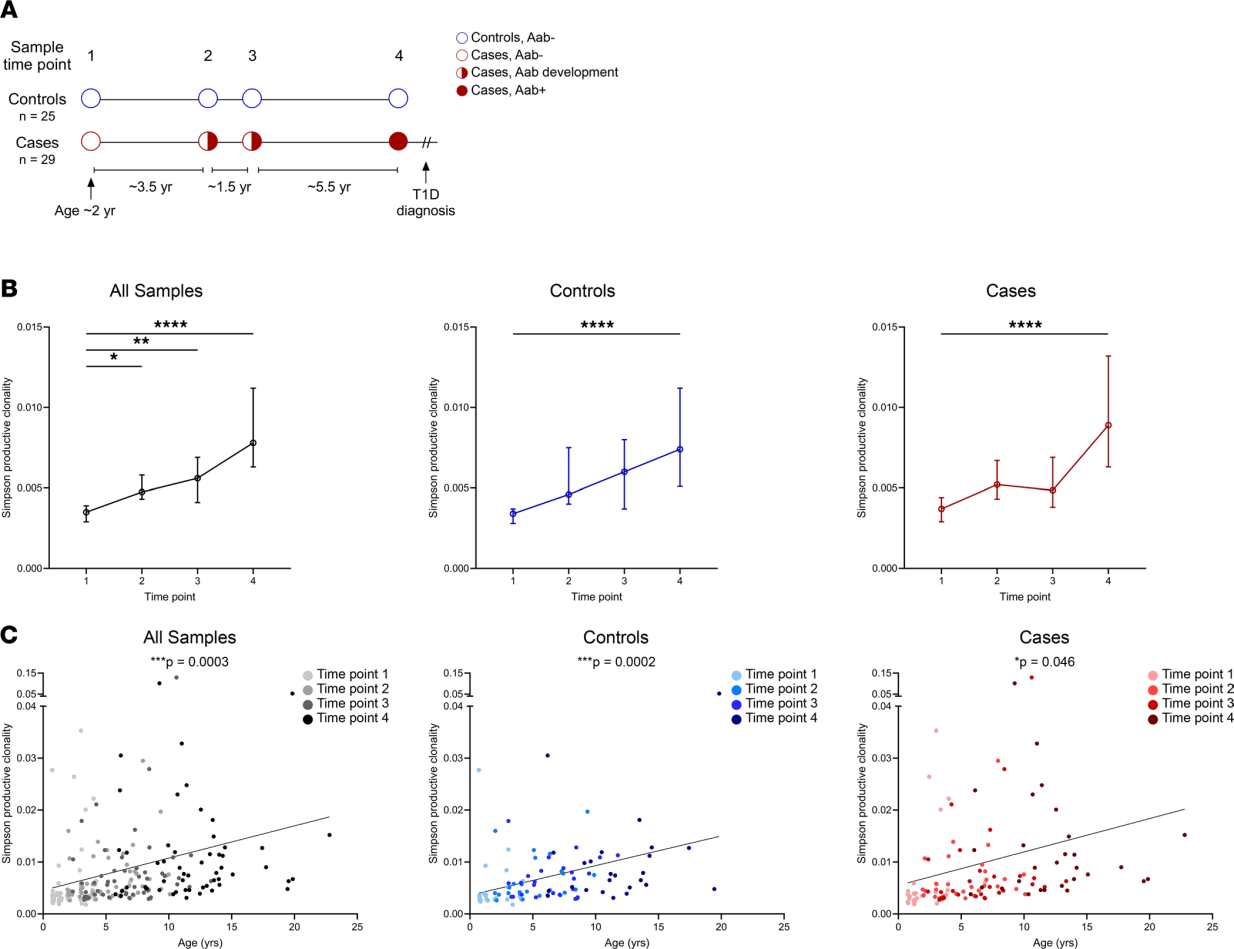
The TCR-β chain repertoire is diverse early in life and contracts with age. (**A**) Study design showing the time points and mean ages at which TCR-β chain sequencing was performed for nondiabetic controls (blue, *n =* 25) and cases (red, *n =* 29) who went on to develop type 1 diabetes, with islet autoantibody-negative visits as open circles, seroconversion as half-filled circles, and autoantibody-positive visits as filled-in circles. (**B**) Plots showing Simpson productive clonality at each time point for all samples (black, left), controls (blue, middle), and cases (red, right). Depicted are medians with 95% CI. (**C**) Scatterplots of Simpson productive clonality relative to age (years) for all samples (black, left), controls (blue, middle), and cases (red, right), with darker colors indicating later time points. *P* values were calculated using mixed-effects models to account for multiple measurements and comparisons for clonality at time point 1 versus all other time points; linear regression was used to compare clonality versus age. *P < 0.05, **P < 0.01, ****P < 0.0001.

To gain a better understanding of the TCR repertoire early in life and during the stages of T1D development, we deep sequenced TCR-β chain repertoires from participants in the Diabetes Autoimmunity Study in the Young (DAISY), an established prospective birth cohort study following genetically at-risk children for the development of T1D-associated autoantibodies (islet autoantibodies) and clinical diabetes (15). Individuals in our nested case-control study were age and sex matched, and the vast majority of cases and controls carried 1 or both of the T1D human leukocyte antigen (HLA) class II risk alleles DQ8 and/or DQ2, with no statistical differences between the groups (Table 1 and Supplemental Data Set 1; supplemental material available online with this article; https://doi.org/10.1172/jci.insight.161885DS1). Cases (*n =* 29) progressed to clinical T1D and were sampled at 4 time points: early in life (mean age 2.1 years), prior to islet autoantibody seroconversion (5.5 years), after seroconversion (6.7 years), and immediately prior to T1D onset (12.2 years). Controls (*n =* 25) were sampled at comparable age-matched time points but did not develop islet autoantibodies or T1D (Figure 1A and Table 1). Importantly, controls and cases had similar numbers of TCR-β sequences at each time point (Supplemental Figure 1, A and B), with more than 50 million total sequences obtained. When analyzing the repertoires, the TCR-β sequences became more clonal over time (Figure 1B), and the Simpson productive clonality metric directly correlated with age in both cases and controls (Figure 1C). There was no difference in Simpson productive clonality between cases and controls at any single time point (Supplemental Figure 2).

Taken together, these results indicate that, early in life, TCR-β repertoires are diverse and become more narrowed with age, likely due to childhood infections and vaccinations resulting in clonal expansion of the responding T cells.

### Distinct TCR V gene usage early in life identifies a subset of T1D cases.

**Figure 2:**
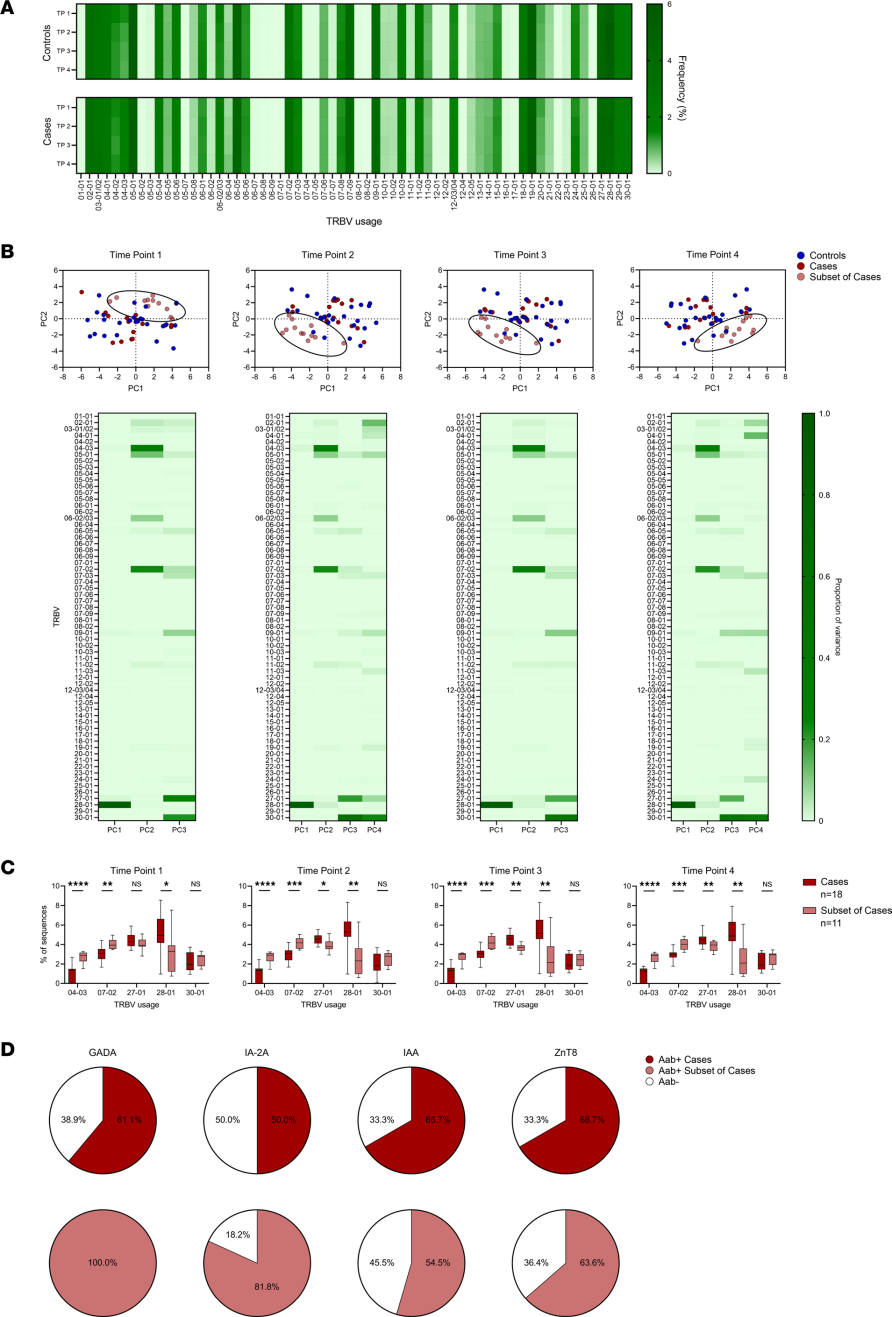
TCR-β V gene usage remains consistent throughout childhood. (**A**) Heatmaps of TCR-β V gene usage at the 4 time points in controls (top) and cases (bottom), with darker green indicating a higher frequency of a given gene. (**B**) Plots of principal component analyses depicting Vβ gene usage by individual at the 4 time points for controls (blue, *n =* 29), cases (dark red *n =* 18), and a subset of cases (light red, *n =* 11). Ellipses denote the same subset of cases throughout the 4 time points. Below each PCA plot is a heatmap quantifying the principal components (Vβ genes) contributing to the variance of each plot, with darker green indicating a higher proportion of the variance. (**C**) Box-and-whisker plots displaying Vβ gene usage accounting for the highest proportion of variance by cases (dark red, *n =* 18) and the subset of cases (light red, *n =* 11). The black center line denotes the median value (50th percentile), while the black box contains the 25th to 75th percentiles of the data set. The black whiskers mark the 10th and 90th percentiles. (**D**) Pie graphs showing the percentage of cases (dark red, top, *n =* 18) and the subset of cases (light red, bottom, *n =* 11) who developed each of the 4 islet autoantibodies tested: glutamic acid decarboxylase autoantibodies (GADA), tyrosine phosphatase–related islet antigen-2 autoantibodies (IA-2A), insulin autoantibodies (IAA), and zinc transporter 8 autoantibodies (ZnT8). Percentages of individuals in each group who were negative for each autoantibody are indicated in white. Time points in cases: 1, early in life; 2, before islet autoantibody positivity; 3, after islet autoantibody positivity; and 4, visit prior to clinical T1D diagnosis. Controls were age matched to cases at each time point. *P* values were calculated using Mann-Whitney *U* tests for Vβ gene usage in cases compared with the subset of cases at each time point. *P < 0.05, **P < 0.01, ***P < 0.001, ****P < 0.0001.

Next, we analyzed the repertoires for differences between controls and cases, beginning with overall V gene usage by the TCR-β chains. Remarkably, V gene usage in the total repertoire was nearly identical at the 4 time points within and between each group (Figure 2A). At the individual level, V gene usage was heterogeneous but remained consistent within any given individual over time (Supplemental Figure 3, A–D). Controls and cases were well matched for HLA class I and II alleles (Supplemental Figure 4, A and B), and this may contribute to the remarkable similarities in V gene usage between the groups. Although V gene usage did not differ between the groups over time, a subset of cases (*n =* 11) could be distinguished from controls (*n =* 25) and remaining cases (*n =* 18) by principal component analyses (PCA) based on percentages of V genes used in the total repertoire (Figure 2B). The variance accounting for the separation of these cases was driven by 5 TCR-β V genes: 28-01 (>88% of PC1), 04-03 (>28% of PC2), 07-02 (>22% of PC2), 27-01 (>17% of PC3), and 30-01 (>22% of PC3 and >23% of PC4; Figure 2B). As such, there were statistically significant increases in the use of Vβ chains 04-03 and 07-02, and there was a decrease in 28-01 that could be identified early in life and persisted over time in a subset of genetically at-risk children who developed T1D (Figure 2C).

Clinically, these cases who had the distinct V gene usage patterns were not different in sex, race, age at islet autoantibody seroconversion, or age at clinical T1D diagnosis compared with the remaining cases (Supplemental Figure 5, A and B). Furthermore, the subset of cases was also well matched for HLA class I and II alleles when compared with the remaining cases (Supplemental Figure 6, A and B), indicating that HLA allele differences were unlikely to segregate the subset of cases. However, there were notable differences in their islet autoantibody profiles. Individuals in the subset of cases were more likely to develop autoantibodies directed against GADA compared with the remaining cases (100% versus 61%, *P =* 0.026), trended toward being more likely to develop tyrosine phosphatase–related IA-2A (82% versus 50%, *P =* 0.126), and were more likely to develop both GADA and IA-2A (82% versus 28%, *P =* 0.008; Figure 2D and Supplemental Figure 7). These differences were not observed for development of insulin autoantibodies (IAA) (55% versus 67%, *P =* 0.697) or ZnT8 autoantibodies (64% versus 67%, *P =* 1.0, Figure 2D).

Overall, there was not a single dominant TCR-β V gene that differentiated cases and controls over time; however, PCA of TCR-β V genes identified a subset of cases early in life that eventually developed distinct islet autoantibody profiles and clinical diabetes.

### Influenza-reactive TCR sequences are present during childhood.

**Figure 3:**
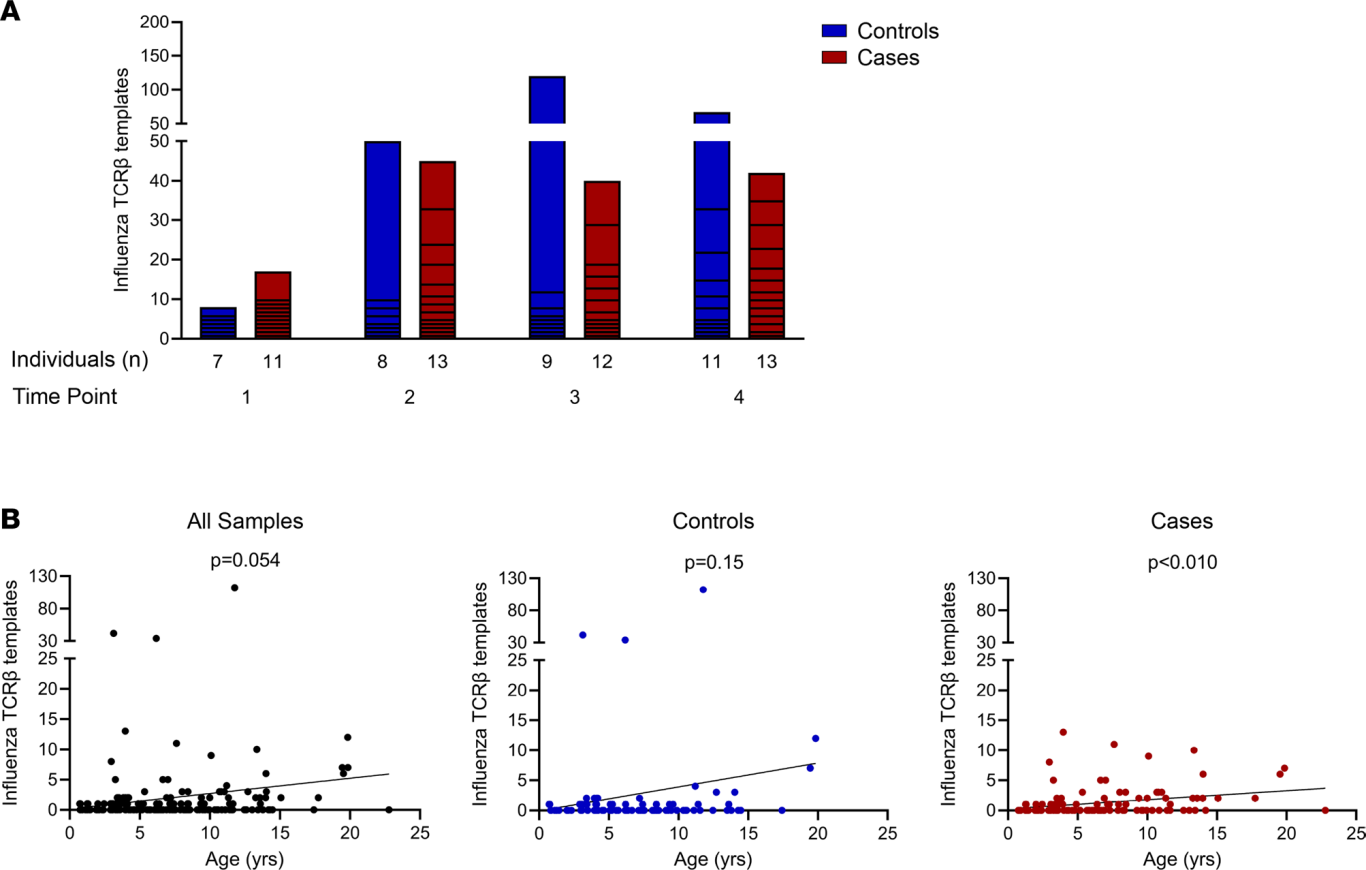
Presence of influenza-reactive TCR-β chain sequences correlates with increasing age. All TCR-β chain templates across individuals and time points were searched for the presence of 6 known influenza-responsive TCR-β chains (identical V, J, and CDR3 amino acid sequences), with 3 sequences identified in our data set. (**A**) Bar graphs showing influenza TCR-β chain template numbers in controls (blue) and cases (red) at the 4 time points. Time points in cases: 1, early in life; 2, before islet autoantibody positivity; 3, after islet autoantibody positivity; and 4, visit prior to clinical T1D diagnosis. Controls were age matched to cases at each time point. (**B**) Scatterplots of influenza TCR-β chain template numbers relative to age (years) for all samples (black, left), controls (blue, middle), and cases (red, right). *P* values were calculated using linear regression for template number versus age.

Influenza A virus is a common pathogen with initial exposure during childhood, and we aimed to use our database of TCR-β sequences to detect antigen-specific sequences for influenza. Using 6 influenza-reactive αβTCRs, 3 CD4 sequences, and 3 CD8 sequences, with known specificities to hemagglutinin or the matrix protein of the virus and restricted to diabetes-risk HLA molecules (16–18), we searched all 4 time points for the presence of the influenza TCR-β chains (identical V, J, and CDR3 amino acid sequences). Three of these sequences were identified comprising hundreds of templates in numerous individuals (Supplemental Table 1). A similar number of cases and controls had influenza sequences at each time point, and TCR-β template numbers in both groups were comparable (Figure 3A). As expected, influenza-reactive TCR-β chain presence and template number correlated with increasing age in both cases and controls with a trend toward statistical significance (Figure 3B). Next, we examined whether a given influenza TCR-β with multiple identical amino acid templates came from different DNA clonotypes. There were 18 samples that had more than 3 identical amino acid sequences for a single influenza TCR-β, and in all of these samples there were multiple DNA clonotypes that converged to a single amino acid TCR-β sequence. This indicates that there was in vivo antigen presentation and expansion of these T cell clones, as opposed to multiple amplifications of a single nucleotide sequence during the polymerase chain reaction used for sequencing.

In summary, we are able to search our TCR database and identify β chain sequences used by influenza-specific T cells, thus providing a proof of concept to detect antigen-specific TCR-β sequences.

### Pancreatic islet–derived CD4 TCRs responding to insulin are more frequent in those who develop diabetes.

**Figure 4:**
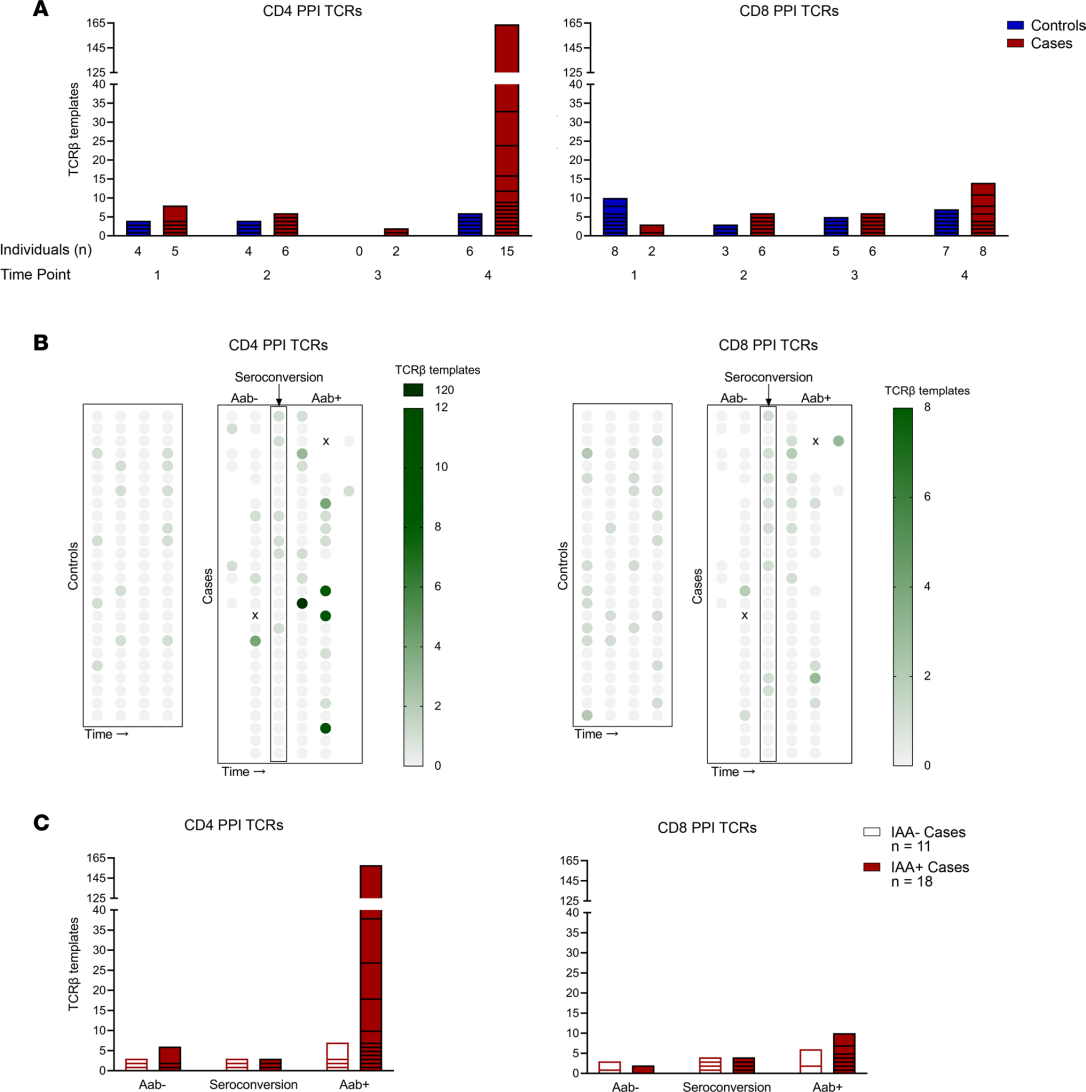
Preproinsulin-reactive TCR-β chain sequences are enriched during progression to type 1 diabetes. All TCR-β chain templates across individuals and time points were searched for the presence of 44 known preproinsulin-responsive (PPI-responsive) TCR-β chains (identical V, J, and CDR3) with 15 sequences identified in our data set. (**A**) Bar graphs showing template numbers of CD4 (left) and CD8 (right) PPI TCR-βs in controls (blue) and cases (red) by time point. Time points in cases: 1, early in life; 2, before islet autoantibody positivity; 3, after islet autoantibody positivity; and 4, visit prior to clinical T1D diagnosis. Controls were age matched to cases at each time point. (**B**) Multivariable plots displaying CD4 (left) and CD8 (right) PPI TCR-β template numbers in controls and cases, with visits aligned by islet autoantibody seroconversion in cases. Darker green indicates a higher template number. (**C**) Stacked bar graphs depicting CD4 (left) and CD8 (right) PPI TCR-β templates in cases who developed insulin autoantibodies (IAA^+^, red) and those who remained insulin autoantibody negative (IAA^–^, white), aligned by timing of seroconversion to any of the 4 islet autoantibodies.

With the ability to detect and quantitate viral-specific TCR-β chains, we next aimed to identify self-antigen–specific TCR sequences during the course of T1D development. Insulin is a major self-antigen in T1D, and its precursor, PPI, has been identified as a target for T cells within the inflamed pancreatic islets of T1D organ donors. To date, 44 PPI-reactive αβTCRs, 14 CD4, and 30 CD8, have been identified from the residual islets of multiple T1D donors (12, 14, 19–22). To determine whether these TCR-β chains were differentially expressed in cases and controls, we searched all 4 time points for the presence of the PPI TCR-β chains (identical V, J, and CDR3). A total of 15 PPI TCR-β chains were found throughout the time points in both groups (6 CD4 and 9 CD8 sequences; Figure 4A and Table 2). Notably, a larger proportion of cases had CD4 PPI TCR-β chain sequences compared with controls (72% versus 36%, *P =* 0.0126; Figure 4A), and the number of these templates was much higher in cases (180 versus 14 for cases and controls respectively, *P <* 0.001). This was not the case for CD8 PPI TCR-β sequences, which were present in similar proportions between cases and controls (52% versus 64%, *P =* 0.417; Figure 4A) and template numbers (29 versus 25 for cases and controls, respectively).

Since islet autoantibodies precede the development of clinical T1D (23), we investigated the timing of the appearance of pancreatic PPI TCR-β sequences in the peripheral blood in relation to islet autoantibody seroconversion. CD4 and CD8 PPI TCR-β sequences were present before or at seroconversion in a few individuals (Figure 4B); however, CD4 PPI TCR-β templates increased considerably after the development of islet autoantibodies, especially when compared with controls at similar time points (Figure 4B). Since CD4 T cells undergo cognate interactions with B cells that can lead to antibody production, we investigated the presence and timing of CD4 PPI TCR-β templates in cases with and without IAA. Cases with IAA (*n =* 18) expressed more CD4 PPI TCR-β templates compared with IAA^–^ cases (*n =* 11), 167 versus 13 (*P <* 0.001, Figure 4C), respectively. Furthermore, the CD4 PPI TCR-β templates primarily accumulated after seroconversion to any islet autoantibody (Figure 4C). Four of the cases with IAA had 8 or more identical amino acid sequence templates for a single CD4 PPI TCR-β, and we examined whether different DNA clonotypes gave rise to these templates. Case 22 had 11 templates of a single CD4 PPI TCR-β that originated from 3 different DNA clonotypes (Supplemental Data Set 1), indicating in vivo presentation and expansion of this T cell clone. In contrast to the CD4 PPI TCR-β sequences, the frequency and accumulation of CD8 PPI TCR-β chains was not as robust as that for CD4 in cases with or without IAA (Figure 4C).

Taken together, these results demonstrate that cases who develop T1D more frequently expressed pancreatic islet–derived CD4 PPI TCR-β sequences compared with controls and had higher numbers of these TCR-β chains. Additionally, IAA^+^ cases expressed significantly more CD4 PPI TCR-β templates after autoantibody seroconversion compared with IAA negative cases, indicating a potential role for CD4 PPI TCRs in the affinity-matured antibody responses to insulin and B cell antigen presentation of PPI to these CD4 T cells.

### Clusters of PPI-reactive TCR-β chains clonally expand prior to diabetes diagnosis.

**Figure 5:**
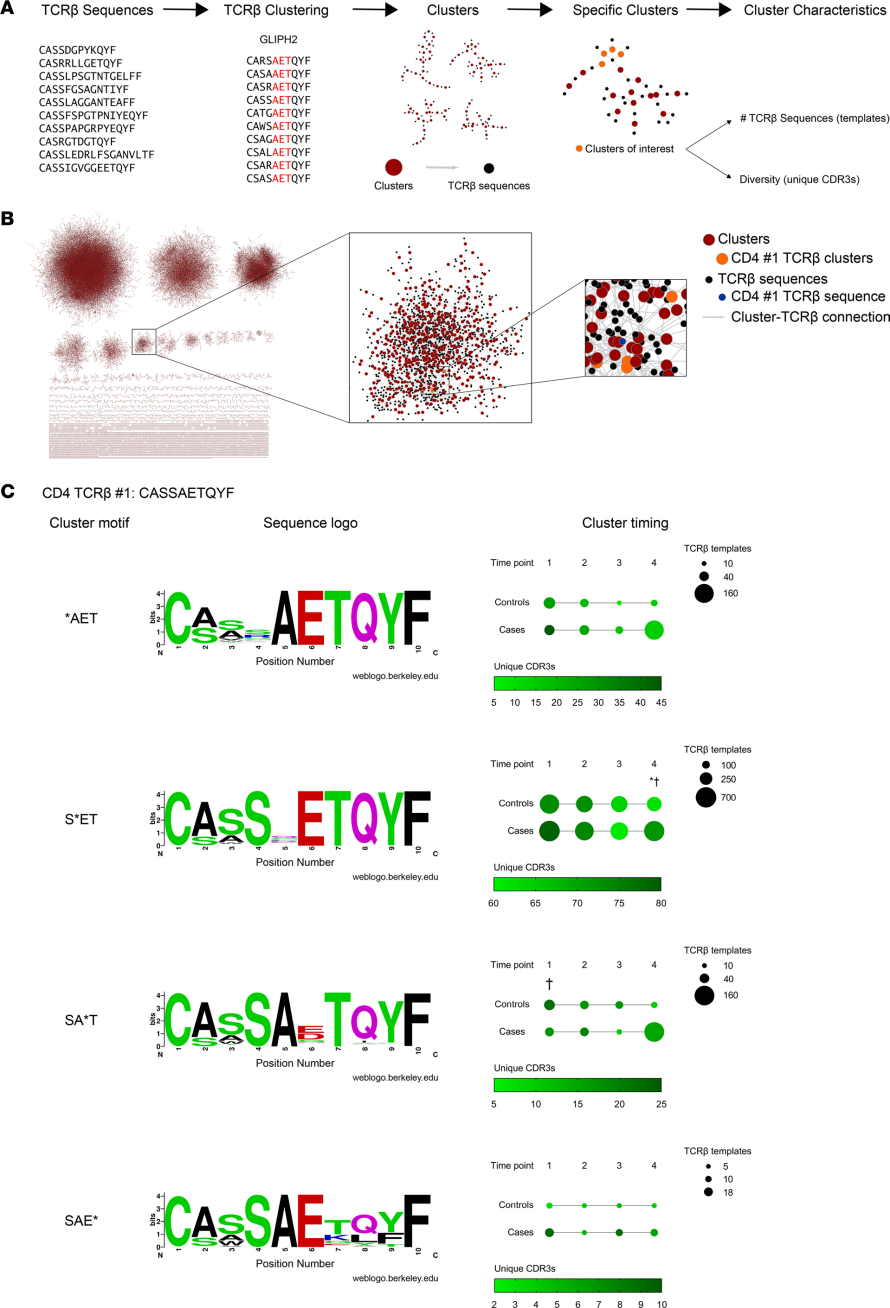
Clusters of TCR-β chain sequences, predicted to recognize similar antigens to preproinsulin, expand during diabetes development. (**A**) Schematic depicting the clustering of TCR-β sequences and subsequent characterization of identified clusters containing preproinsulin-reactive TCR-β chains. (**B**) A representation of TCR-β chain sequence clustering from a single individual at 1 time point generated using the GLIPH2 program; depicted are 68,639 total clusters from case #16 at time point 3. TCR-β chain sequences (black) are grouped into clusters (red) based upon predictions to bind similar peptides. CD4 TCR #1 (blue) that recognizes insulin B chain amino acids 9–23 were found in 4 TCR clusters (orange) at this time point. (**C**) Sequence logos for the 4 clusters that contain the PPI-reactive CD4 TCR #1 showing the frequency of amino acids at each position within the CDR3β sequences (left). Larger letters indicate a higher prevalence of an amino acid at a particular position. Multivariable plots in panels depict the 4 cluster motifs at each time point in controls and cases (right). Dot size indicates the number of TCR-β chain templates composing the cluster, while a darker green color depicts a higher number of unique CDR3β sequences in the cluster (a measure of TCR diversity). Time points in cases: 1, early in life; 2, before islet autoantibody positivity; 3, after islet autoantibody positivity; and 4, visit prior to clinical T1D diagnosis. Controls were age matched at each time point. *P* values were calculated using mixed-effects models to account for multiple measurements and comparisons between controls and cases at each time point for either template number (*) or CDR3β diversity (†). *P < 0.05, †*P <* 0.05. Supplemental Table 2 provides full statistics for temporal changes within a cohort and for comparisons between cases and controls.

Although pancreatic PPI TCR-β chains were found in cases and controls, the overall numbers were much lower than that for influenza A–reactive TCR-β chains, despite having identified 15 shared PPI TCR-β chain sequences compared with only 3 for influenza. Therefore, we sought to identify other TCR-β chains that may recognize similar or nearly identical antigens as the identified PPI-reactive TCRs. Within αβTCRs, each chain contains a unique complementarity determining region 3 (CDR3), which encodes for the portion of the TCR that is most directly in contact with the antigen. The clustering program Grouping of Lymphocyte Interactions by Paratope Hotspots (GLIPH) analyzes TCR sequences and groups TCRs based upon a number of factors including HLA alleles, V gene usage, CDR3 sequence, and CDR3 length (24). Thus, the algorithm generates clusters of TCRs that may recognize shared or similar antigens to a parent TCR sequence. We clustered TCR-β sequences within an individual at each of the 4 time points using GLIPH2 (Figure 5A) (25). Tens of thousands of TCR-β clusters were identified in a single individual at 1 time point (Figure 5B), and we narrowed our focus to those clusters that included a known PPI TCR-β sequence. The most frequent PPI TCR-β found in this study, termed CD4 TCR #1, recognizes the well-characterized insulin B chain amino acids 9-23 peptide (B:9-23, Table 2) (26–30). After clustering all of the samples, 4 cluster motifs were identified which contained CD4 TCR #1 and additional, nearly identical TCR-β sequences (Figure 5C). From these TCR-β sequences, a single CDR3 motif was determined for each of the 4 clusters (i.e., sequence logo) (Figure 5C). Although the 4 clusters consisted of TCR-βs with nearly identical CDR3 sequences, each CDR3 motif contained 1 position, indicated by an asterisk, with varied amino acids at that particular position. As such, all TCR-β sequences within a given cluster shared 1 CDR3 motif, with variability among the TCR-β chain CDR3 sequences at the indicated positions. Importantly, these clusters containing the related TCR-β chains could be found at additional time points in those who expressed the parent PPI TCR-β, as well as in individuals who did not express the parent sequence.

Among these 4 clusters, the temporal dynamics differed between cases and controls. The *AET cluster contained similar numbers of templates for both controls and cases at time point 1 (Figure 5C, first row), but templates decreased in controls and expanded in cases over time with a trend toward statistical significance (*P =* 0.065; Supplemental Table 2 for statistical comparisons using mixed effects models to account for multiple measurements and comparisons). The S*ET cluster dynamics were again similar in cases and controls until time point 4, in which there were more templates and CDR3 sequences in cases compared with controls (Figure 5C, second row). Interestingly a third cluster, SA*T, contained more unique CDR3s at time point 1 in controls that decreased over time, along with template numbers, indicating a contraction of this cluster repertoire (Figure 5C, third row). In contrast, this SA*T cluster in cases stayed relatively stable in terms of CDR3 diversity, but template numbers increased over time, signifying a clonal expansion. The fourth cluster, SAE*, was found infrequently in both groups (Figure 5C, fourth row). Next, we analyzed all samples for clusters containing the second and third most frequent PPI TCR-βs, CD4 #2 and CD8 #7 (Table 2). For CD4 #2, 4 motifs were identified, with 1 cluster showing differences in diversity at time point 1 between cases and controls (Supplemental Figure 8 and Supplemental Table 3). There were 6 distinct clusters for CD8 #7 with differences in template number and diversity at specific time points when comparing cases and controls (Supplemental Figure 9 and Supplemental Table 4).

Overall, TCR clustering allowed for the identification and visualization of hundreds of additional TCR-β chain sequences predicted to recognize similar antigens to those as PPI-specific TCR-βs. Several of these clusters preferentially expanded during diabetes development, providing a potentially powerful biomarker to monitor the progression during T1D development.

### Validation of PPI-reactive TCR-β chain sequences and clusters in a new-onset T1D cohort.

Lastly, we aimed to validate our findings from DAISY by performing the same sequencing and analyses in a separate cohort of new-onset T1D patients (*n =* 143). The new-onset cohort was well matched to DAISY cases and controls in terms of sex, ethnicity, HLA risk for T1D, and numbers of TCR-β chains sequenced (Supplemental Table 5 and Supplemental Data Set 1). Importantly, samples from the new-onset individuals were collected on average 5 days (median 2 days) after starting exogenous insulin treatment, such that insulin injections were unlikely to alter the TCR repertoire. We identified 8 of 15 PPI TCR-β sequences in new-onset individuals (2 CD4 and 6 CD8), and both of the most frequently identified PPI TCR-β sequences in DAISY, CD4 #1, and CD8 #7 were also present within multiple individuals in the new-onset cohort (Table 2). Furthermore, we analyzed the new-onset samples expressing the PPI TCR-β chains for the presence of the clusters identified in the DAISY cohort. The 4 clusters from PPI CD4 #1 and the 6 from PPI CD8 #7 were identified in new-onset individuals who expressed the parent TCR-β chain (Supplemental Figure 10, A and B), confirming the DAISY results in another cohort consisting of new-onset T1D patients.

Taken together, these findings reveal that identical V, J, and CDR3 sequences from 8 pancreatic PPI TCR-β chains and clusters around these antigen-specific sequences can be identified in the peripheral blood of those at risk and with newly diagnosed T1D, indicating a continued role for these TCRs throughout disease development.

## Discussion

Relatively little is known about the composition, dynamics, and development of human TCR repertoires early in life and into childhood; however, decades-spanning cross-sectional (6, 7) studies have shown that TCR-β diversity narrows with increasing age, and short-term longitudinal studies (up to 2 years) indicate repertoire stability (31, 32). Our studies now provide a robust longitudinal data set of TCR-β repertoires from children early in life throughout childhood, and we demonstrate that TCR-β repertoire diversity decreases with age while clonality increases, as demonstrated by expansion of influenza A–reactive TCR-β sequences. Importantly, the notion of clonal expansion over time is applicable to T cells specific to the self-antigen insulin, as these TCR-β sequences were enriched in individuals developing T1D. Understanding TCR repertoire changes early in life is exceedingly important for delineating the role of T cells in diseases that present during childhood, including T1D.

By sequencing the TCR-β repertoire from peripheral blood in children at risk for T1D, we demonstrate that, based upon Vβ gene usage early in life, a subset of children could be identified who later developed distinct islet autoantibody profiles with antibodies directed against GAD and IA-2A followed by clinical diabetes. Similarly, in a recent longitudinal study from children genetically at risk for T1D, gene expression analyses identified differences in peripheral blood NK cell and memory CD4 T cell transcriptome signatures that correlated with the development of initial islet autoantibody specificity and clinical diabetes onset (33). Furthermore, specific gene signatures alongside patterns of islet autoantibodies tracked with progression to T1D at an individual level. These results and our findings indicate that changes in immunological signatures, transcriptome profiling, and TCR-β chain sequencing during early childhood may help delineate individuals who are more likely to develop islet autoantibodies and progress to clinical T1D.

In general, the TCR repertoire within a given individual is primarily composed of private TCRs and minimally overlaps with those of others (5, 34–36). However, advances in next-generation sequencing have allowed for deep sequencing of the TCR repertoire from more individuals, resulting in the identification of specific sequences that are shared between individuals and associated with different disease states (37–39). We were able to identify both known influenza A–reactive and PPI-responding TCR-β chains in our data set. We focused on PPI-reactive T cells, as these have been well characterized from the pancreatic islet infiltrates of T1D organ compared with T cells directed against GAD and IA-2A (12, 14, 19–22), despite finding specific Vβ genes early in life associated with a subset of cases that developed GAD and IA-2A autoantibodies. Of the 44 PPI-reactive TCR-β chains identified from pancreatic islets of T1D organ donors, we show that the exact V, J, and CDR3 from 15 of 44 sequences (34%) are shared in the peripheral blood of individuals genetically at risk for T1D. Additionally, 8 of the 15 insulin-reactive TCR-β chains were found in a separate cohort of new-onset T1D patients, indicating a continued role for these TCRs throughout disease development. The lower number of TCR-β sequences in our new-onset cohort may be indicative of some TCRs being more involved earlier in the disease process, prior to clinical symptoms. Alternatively, there may be more β cell destruction in new-onset individuals compared with those in the preclinical stages of T1D, as evidenced by a recent study demonstrating that proliferation of proinsulin-specific CD4 T cells in children with new-onset T1D correlated to residual β cell function (40).

Interestingly, CD4 PPI–reactive TCR-β sequences were more prevalent than PPI CD8 sequences in the peripheral blood prior to clinical T1D and in our new-onset cohort. It is appreciated that HLA class I is overexpressed on the residual insulin containing pancreatic islets from T1D organ donors (41), and CD8 T cells are the more abundant T cell subtype infiltrating pancreatic islets (11, 13, 21). Thus, it is plausible that the CD8 PPI–reactive TCR-β sequences studied here may have been more localized to the pancreas and not as prevalent in the peripheral blood as compared with PPI CD4 TCR-β chain sequences.

It should be noted that our study focused on TCR-β sequencing, and as sequencing technology continues to advance, it will be important to deep sequence both TCR α and β chains, including paired sequences from a large number of single T cells. Having paired α and β chains from individual T cells would then allow for confirmation of antigen specificity of a given TCR, such as those identified in our clustering analyses. Undersampling of the vast TCR repertoire is also a limitation, and future studies should focus on deeper sequencing TCR-β chains. The potential exists to target specific Vβ genes and to deep sequence those TCR-β chains to identify more individuals with a given receptor of interest, such as those from PPI-reactive T cells in individuals progressing through the stages of T1D.

Importantly, we demonstrate that clusters containing TCR-β chains predicted to bind PPI peptides could be found at additional time points in individuals who expressed the parent TCR-β chain, as well as at multiple time points of those who never expressed it, thus allowing for a visualization of disease-related TCR numbers and CDR3 diversity over time. One advantage of sequencing and clustering TCRs is that it obviates the need for functional T cell assays to monitor disease-relevant cells. Although informative, functional T cell assays (e.g., fluorescent peptide-MHC multimer staining, proliferation assays with fluorescent cell staining dyes, and cytokine enzyme linked immunospot [ELISpot] assays) are often challenging to perform due to variables such as the necessity for large blood volumes, the need for living cells, and biases that may occur due to operator use.

While sequencing TCRs directly from DNA has advantages, cell phenotype information cannot be obtained. However, Bonifacio and colleagues longitudinally examined the gene expression profiles of CD4 T cells that proliferated to proinsulin and GAD65 beginning early in life from children at risk for T1D (42). Islet-responsive naive CD4 T cells were present in all children at 6 months of age; however, these cells developed a memory phenotype with a predominant inflammatory gene expression profile after islet autoantibody seroconversion. Furthermore, the proinsulin- and GAD-reactive CD4 T cells proliferated more robustly following islet autoantibody development compared with children without autoantibodies. These results support the findings in our study in which PPI CD4 TCR-β sequences and clusters containing these sequences are present early in life and then expand following islet autoantibody seroconversion indicating that preproinsulin- and proinsulin-responsive CD4 T cells are present early and mature during disease progression.

In conclusion, our studies reveal that, in T1D, where disease-relevant TCRs have been obtained from the target organ, sequencing and clustering of related TCR-β chains in the peripheral blood provide insight into disease progression, as there is an enrichment of PPI-reactive sequences during the progression to clinical disease. TCR sequencing may provide a robust and reproducible biomarker during the course of T1D development and aid in the timing and monitoring of therapies for disease prevention. Our results also provide a framework for using TCR sequences in fields of medicine where disease-relevant sequences are known, including autoimmune diseases, infections, and cancers.

## Methods

### Study design.

Subjects were recruited into the DAISY, which is an ongoing prospective birth cohort study following genetically at-risk children and adolescents for the development of islet autoantibodies and clinical T1D (15). Study subjects for the new-onset T1D cohort were recruited from the Barbara Davis Center for Diabetes Clinics. Peripheral blood was obtained for islet autoantibody measurements, HLA typing, and TCR-β sequencing.

### Islet autoantibody measurements and HLA genotyping.

Serum obtained from peripheral blood was used to measure islet autoantibodies to GADA, tyrosine phosphatase–related IA-2A, ZnT8A, and IAA using fluid-phase radio-binding assays as previously described (43, 44). ZnT8A was measured from participants beginning in 2010 and later. Harmonized assays for GADA and IA-2A were available starting in 2010, and index values obtained before this time were converted to DK units. The same positive cut-off values were applied to all participants with an index of 0.010 for IAA, 20 DK units for GADA, 5 DK units for IA-2A, and an index 0.020 for ZnT8A. Previously collected and stored DNA samples from DAISY participants were used to type HLA class I (A, B, and C) and class II alleles (DQA1, DQB1, DRB1, DPA1, and DPB1) using a targeted next-generation sequencing assay with hybrid capture technology following the manufacturer’s instructions (AlloSeq Tx17 from CareDx Lab Solutions Inc.). For the new-onset T1D cohort, HLA typing of the HLA-DRB1, DQA1, and DQB1 alleles was performed using oligonucleotide probes as previously described (45).

### TCR-β chain immunosequencing.

Genomic DNA was extracted from cryopreserved buffy coat samples obtained from peripheral blood following the manufacturer’s instructions using the QIAamp Blood Extraction kit (Qiagen). TCR-β chains were sequenced using the immunoSEQ Assay (Adaptive Biotechnologies) as previously described (4, 46). The identification of specific V and J genes was carried out according to the definitions established by the International ImMunoGeneTics (IMGT) collaboration (47). Raw data processing and sequencing analyses were performed using the immunoSEQ Analyzer 3.0 online platform (http://www.adaptivebiotech.com/immunoseq). The following analyses were performed directly through the immunoSEQ Analyzer 3.0: Simpson productive clonality measurements, number of productive TCR-β templates, and PPI TCR-β searches. The calculations for TCR-β V gene (TRBV) usage were performed in Microsoft Excel after downloading the raw data from the immunoSEQ Analyzer 3.0. Subsequently, the TRBV usage data were then used in the PCA conducted using GraphPad Prism 9.2 (GraphPad Software).

### TCR-β chain clustering algorithm and analyses.

After the sequencing data for each individual at each time point were downloaded from the immunoSEQ Analyzer 3.0 platform, clustering analyses were performed using the GLIPH2 online platform (http://50.255.35.37:8080/) (25). For all clustering, the reference database version 2.0 was selected (reference data set consisting of bulk sequencing data from T cells) with HLA typing included. All samples were first run against the CD4 reference database to identify the CD4 PPI TCR-β clusters, and then all samples were run against the CD8 reference database to identify the CD8 PPI TCR-β clusters. Further analyses of clusters were then performed using Microsoft Excel. Both Cytoscape version 3.8.2 and GraphPad Prism 9.2 software were used to graph the clustering data.

### Data and materials availability.

All immunosequencing data generated in this study are freely available for analysis and download from the Adaptive Biotechnologies immuneACCESS site under the immuneACCESS terms of use (https://clients.adaptivebiotech.com/pub/mitchell-2022-JCII).

### Statistics.

Statistical analyses were performed using R software version 4.1 (R Core Team, Vienna), GraphPad Prism 9.2 (GraphPad Software), and SAS 9.4 (SAS Institute). *P* < 0.05 was considered significant. Continuous variables (age at time points) were compared using 2-tailed *t* tests, and Fisher’s exact test was used for categorical variables (sex, race, ethnicity, presence of islet autoantibodies, proportion of individuals with a given HLA type, proportions having specific TCR-β chain sequences, and TCR-β chain template numbers). Linear regression analyses were used to compare Simpson productive clonality to age. Mann-Whitney *U* tests were used to compare Simpson productive clonality in cases versus controls at each time point and to compare TRBV gene usage, age at seroconversion, and age at onset in cases versus the subset of cases. Mixed-effects models were used to compare groups and time points while accounting for the correlation of multiple measurements within a participant. Tukey’s Honestly Significant Difference test was used to control for multiple testing when performing pairwise comparisons.

### Study approval.

The clinical investigation in this study was conducted in accordance with the Declaration of Helsinki principles, with study approvals provided by the Colorado Multiple IRB. All participants — or participant guardians, if the participant was less than 18 years of age — provided written informed consent.

## Author contributions

AMM, AKS, KMS, MN, PAG, MJR, and AWM designed the studies. AMM, EEB, KAM, KW, and LY performed the research. LP conducted an independent statistical analysis of the study data. AMM, AKS, KMS, MN, PAG, MJR, and AWM wrote the manuscript. AWM is the guarantor of this work, had full access to all the data in the study, and takes responsibility for the data integrity and accuracy of analyses.

## Supplementary Material

Supplemental data

Supplemental data set 1

## Figures and Tables

**Table 1:**
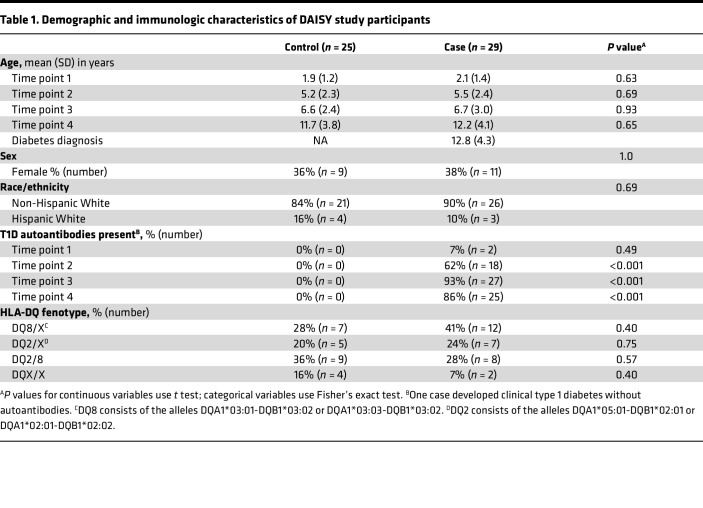
Demographic and immunologic characteristics of DAISY study participants

**Table 2:**
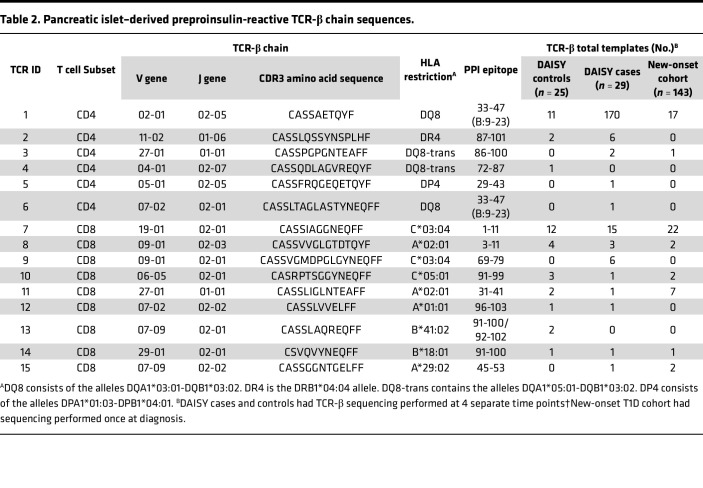
Pancreatic islet–derived preproinsulin-reactive TCR-β chain sequences.

## Version 1 08/23/2022

In-Press Preview

## Version 2 09/22/2022

Electronic publication
