# Supplementary material for: Temporal development of T cell receptor repertoires during childhood in health and disease
Source: JCI Insight. 2022 Sep 22;7(18):e161885. doi: 10.1172/jci.insight.161885 (PMC9675557; doi:10.1172/jci.insight.161885)
Supplement: Supplemental data [file jciinsight-7-161885-s088.pdf]

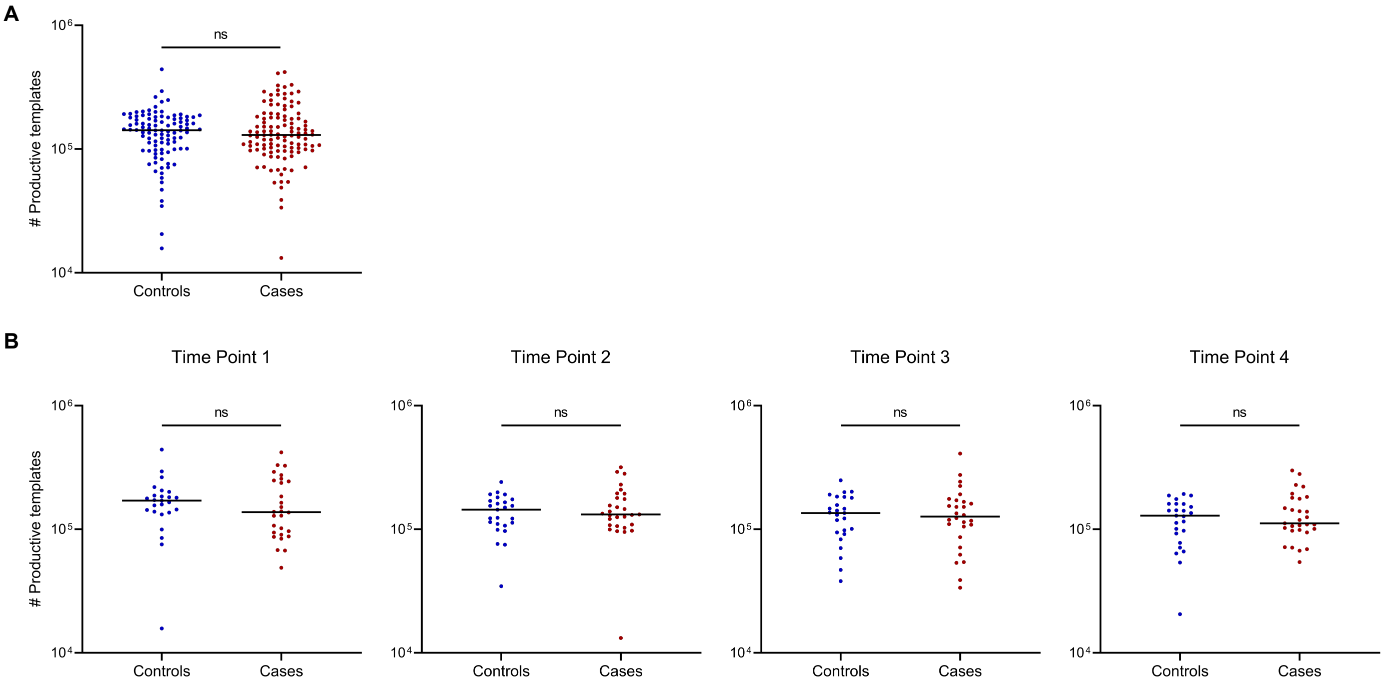

Supplemental Figure 1. Equivalent T cell receptor beta chain sequencing depth in controls and cases across time points. (**A**) Dot plots showing number of productive TCRβ sequences for samples from controls (blue) and cases (red). (**B**) Dot plots displaying number of productive TCRβ sequences per sample for controls and cases at each of the four time points. P-values were calculated using mixed-effects models to account for multiple measurements and comparisons.

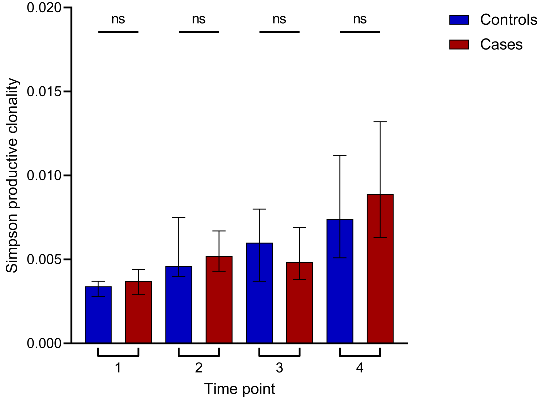

Supplemental Figure 2. T cell receptor beta chain clonality at each time point in controls and cases. Plots showing Simpson productive clonality at each time point for controls (blue) and cases (red). P-values were calculated using Mann-Whitney tests comparing controls to cases at each time point.

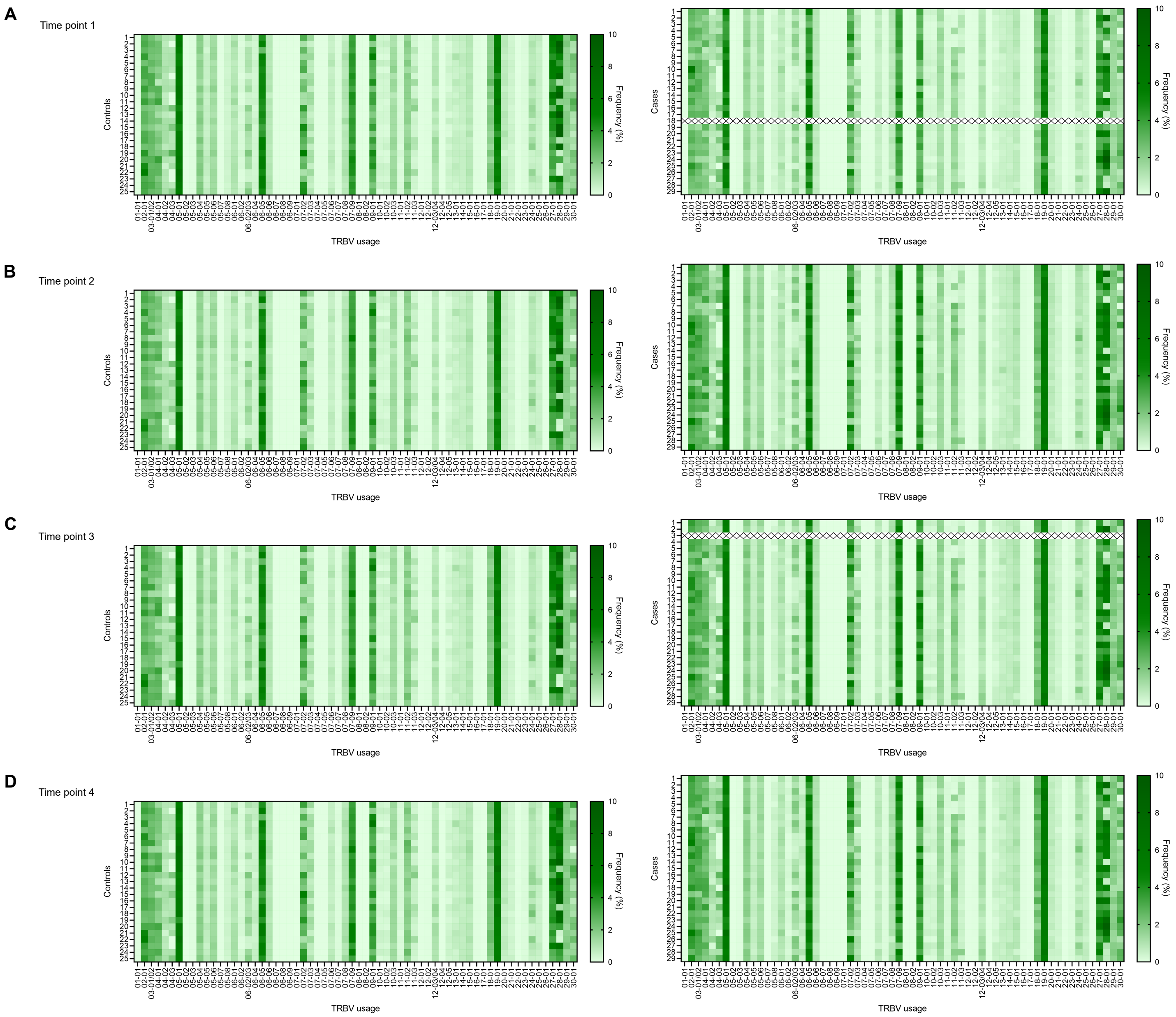

Supplemental Figure 3. Individual-level T cell receptor Vβ gene usage throughout childhood. Heat maps of Vβ gene usage at (**A**) time point 1; (**B**) time point 2; (**C**) time point 3; and (**D**) time point 4 in controls (left) and cases (right), with darker green indicating a higher frequency of a given gene. Individuals are labeled on the y-axis and Vβ genes on the x-axis.

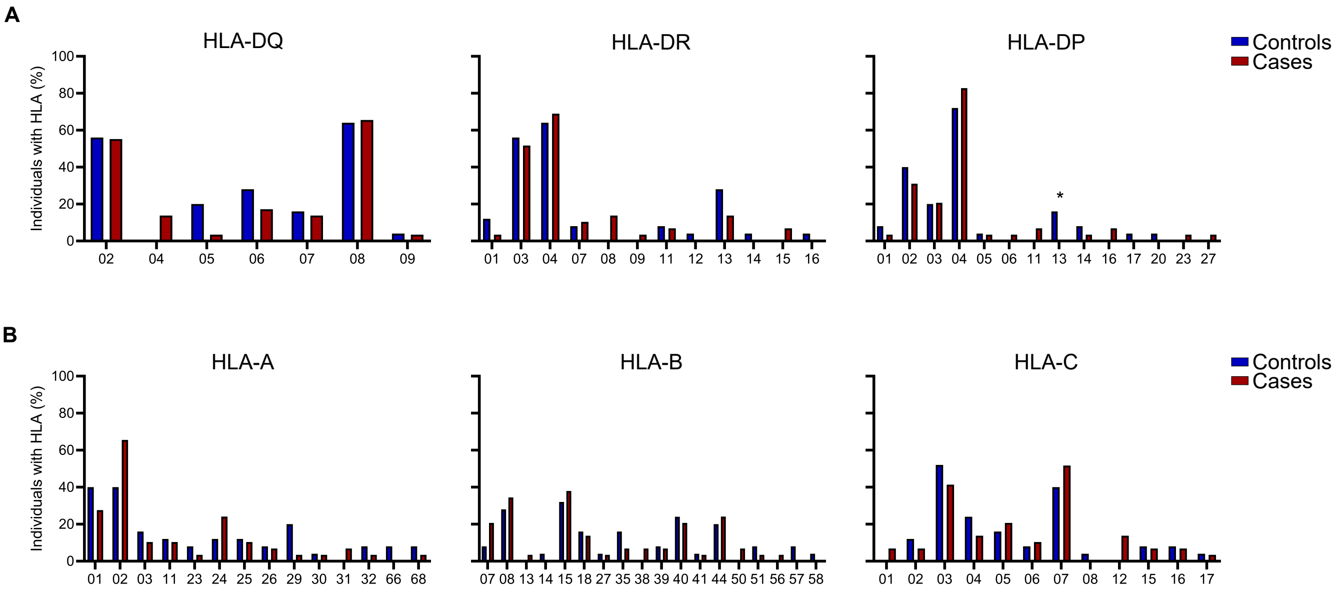

Supplemental Figure 4. HLA class I and II typing in controls and cases. Bar graphs showing class II (A) and class I (B) HLA types in controls (blue) and cases (red). P-values were calculated using Fisher exact tests comparing proportions of controls and cases having each HLA type. *p<0.05.

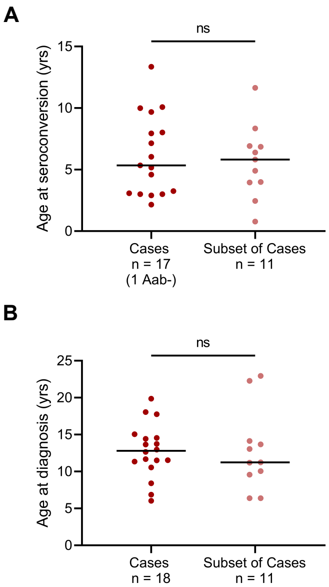

Supplemental Figure 5. Age at islet autoantibody seroconversion and diabetes diagnosis in cases. Dot plots showing (**A**) the age at seroconversion to any islet autoantibody and (**B**) the age at type 1 diabetes diagnosis in cases (dark red, n=18) and a subset of cases identified by principal component analysis of Vβ gene usage early in life (light red, n=11). P-values were calculated using Mann-Whitney tests comparing cases to the subset of cases.

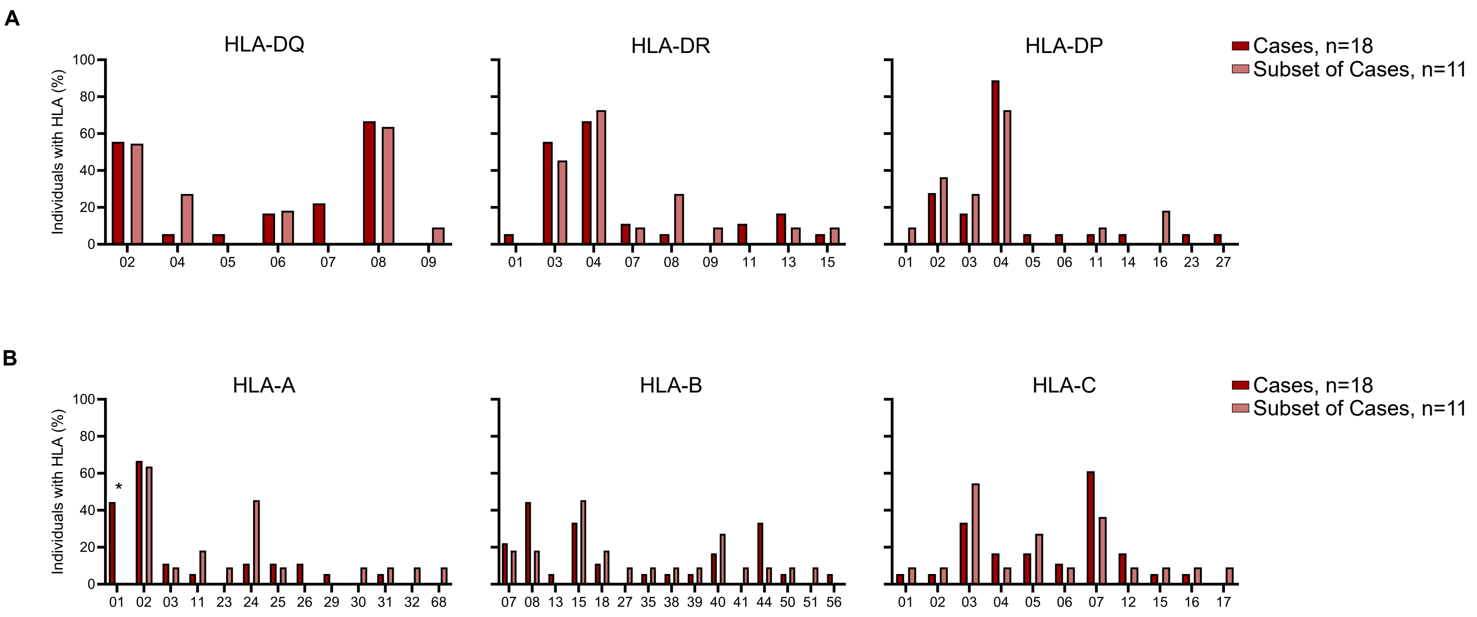

Supplemental Figure 6. HLA typing in cases and a subset of cases with distinct Vβ gene usage early in life. Bar graphs showing class II (**A**) and class I (**B**) HLA types in cases (dark red, n=18) and a subset of cases identified by principal component analysis of Vβ gene usage early in life (light red, n=11). P-values were calculated using Fisher exact tests comparing proportions of controls and cases having each HLA type. *p<0.05.

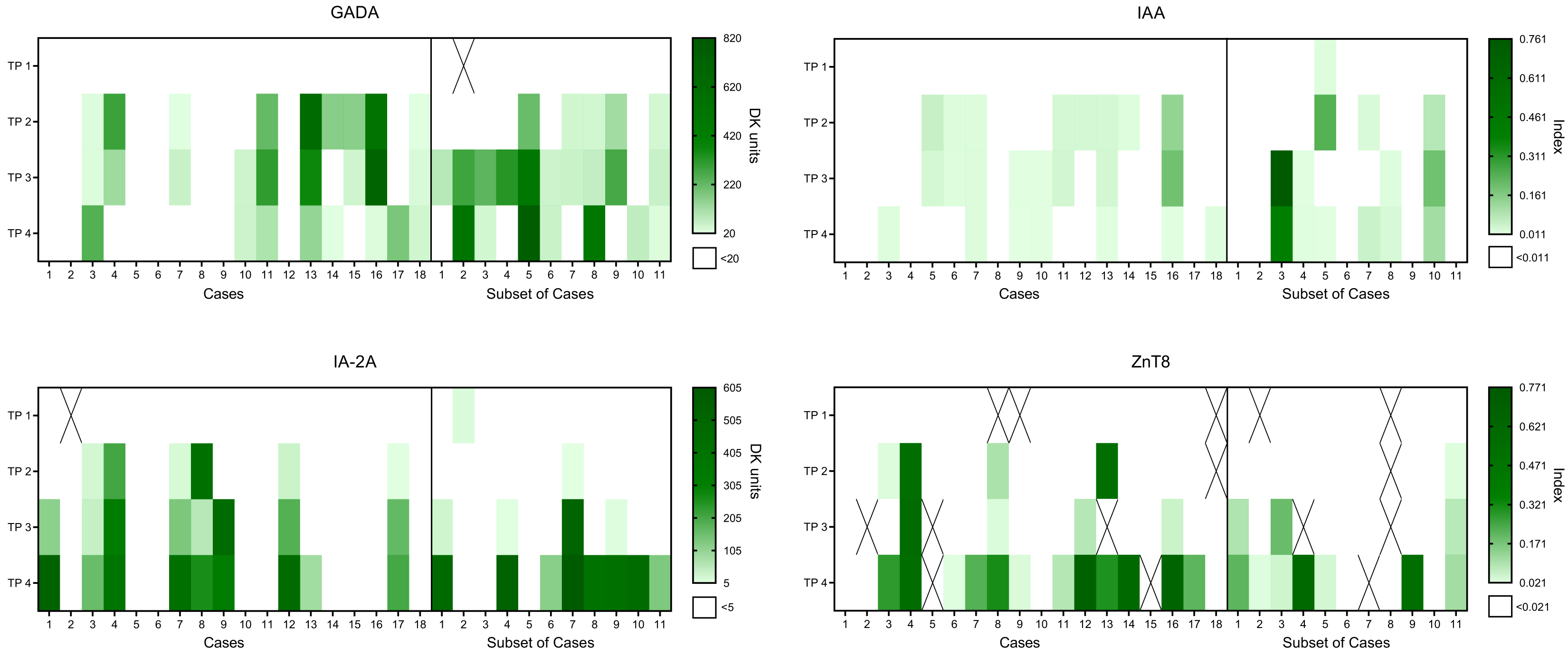

Supplemental Figure 7. Islet autoantibody levels and timing in cases. Heat maps of islet autoantibody levels in cases and the subset of cases identified by principal component analysis of Vβ gene usage early in life, with darker green indicating a higher level of autoantibody. Glutamic acid decarboxylase autoantibodies (GADA), insulinoma-2-associated autoantibodies (IA-2A), insulin autoantibodies (IAA), and zinc transporter 8 autoantibodies (ZnT8) were measured in each individual at the four time points. Time points are labeled on the y-axis and individuals on the x-axis. X indicates an islet autoantibody that was not measured in an individual at a given timepoint.

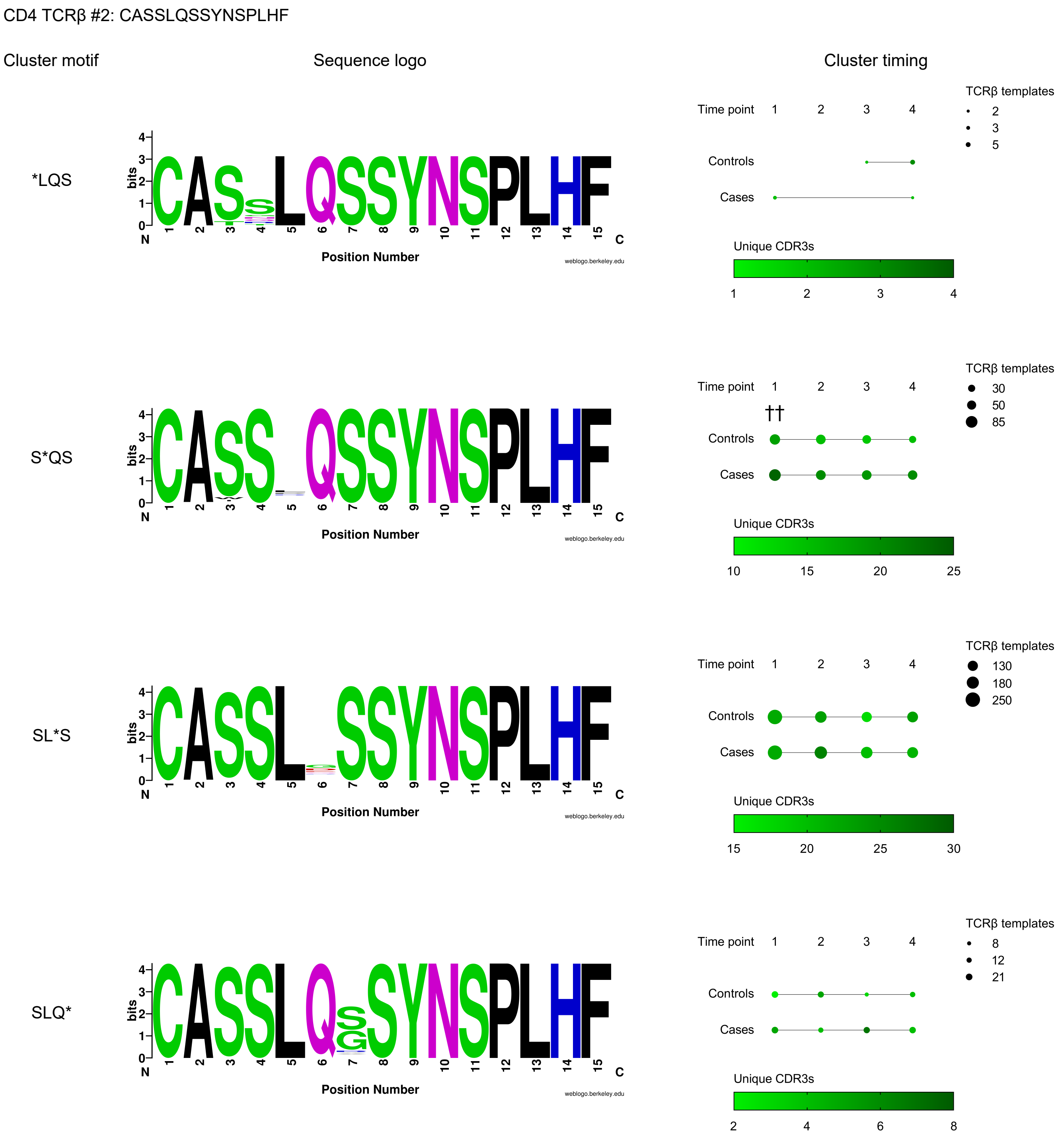

Supplemental Figure 8. Clusters of T cell receptor beta chain sequences predicted to recognize similar peptides as preproinsulin-reactive TCR #2. Sequence logos for the four clusters that contain the PPI-reactive CD4 TCR #2 showing the frequency of amino acids at each position within the CDR3β sequences (left). Larger letters indicate a higher prevalence of an amino acid at a particular position. Multivariable plots in panels depict the four cluster motifs over the four time points in controls and cases (right). Dot size indicates the number of TCRβ chain templates comprising the cluster, while a darker green color depicts a higher number of unique CDR3β sequences in the cluster (a measure of TCR diversity). P-values were calculated using mixed-effects models to account for multiple measurements and comparisons between controls and cases at each time point for either template number (*) or CDR3β diversity (†). *p<0.05, †p<0.05. Table S3 provides full statistics for temporal changes within a cohort and for comparisons between cases and controls.

CD8 TCRβ #7: CASSIAGGNEQFF

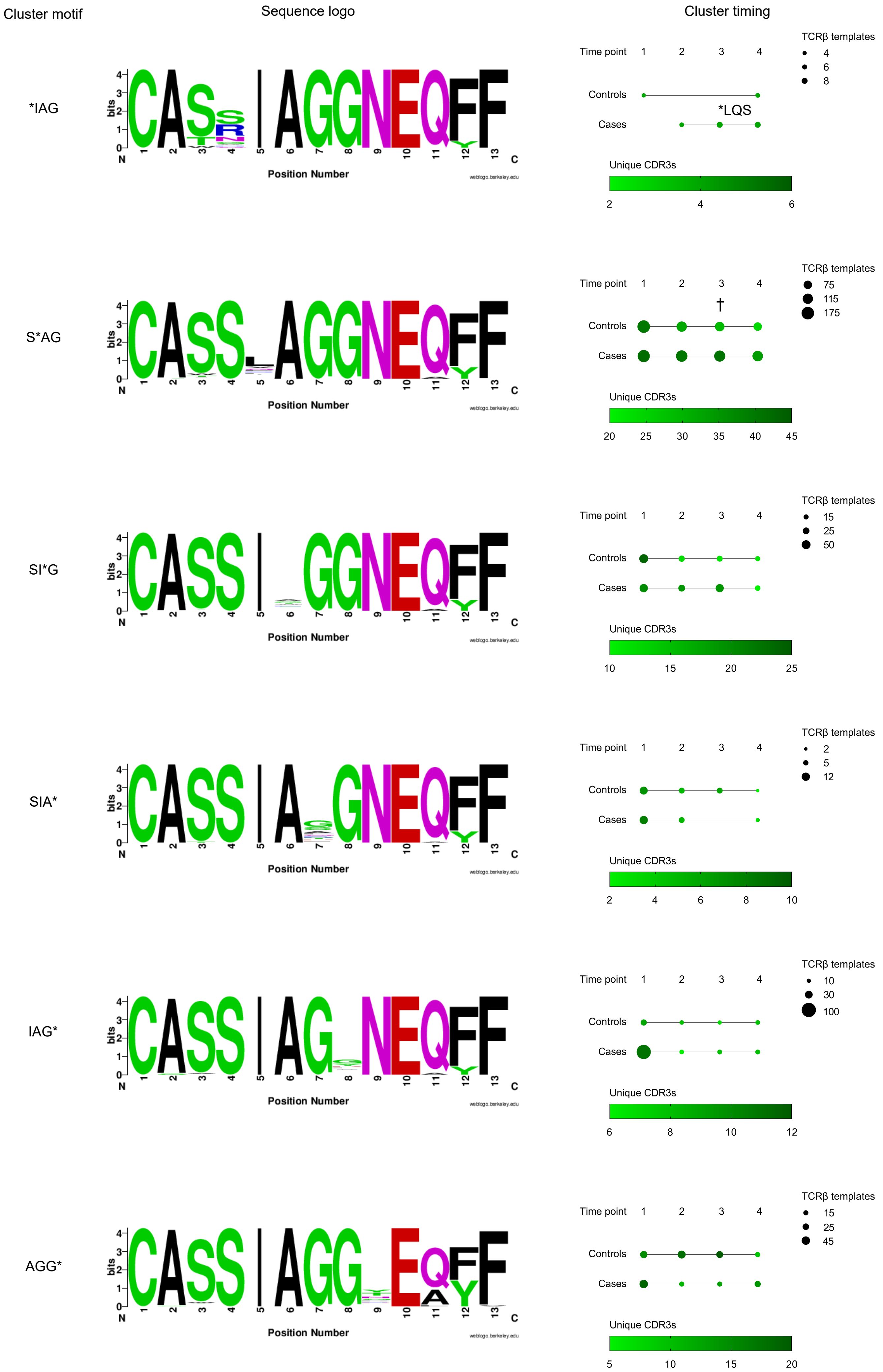

Supplemental Figure 9. Diversity and size of T cell receptor beta chain clusters predicted to bind similar peptides as preproinsulin-reactive TCR #7. Sequence logos for the six clusters that contain the PPI-reactive CD8 TCR #7 showing the frequency of amino acids at each position within the CDR3β sequences (left). Larger letters indicate a higher prevalence of an amino acid at a particular position. Multivariable plots in panels depict the six cluster motifs over the four time points in controls and cases (right). Dot size indicates the number of TCRβ chain templates comprising the cluster, while a darker green color depicts a higher number of unique CDR3β sequences in the cluster (a measure of TCR diversity). P-values were calculated using mixed-effects models to account for multiple measurements and comparisons between controls and cases at each time point for either template number (*) or CDR3β diversity (†). *p<0.05, †p<0.05. Table S4 provides full statistics for temporal changes within a cohort and for comparisons between cases and controls.

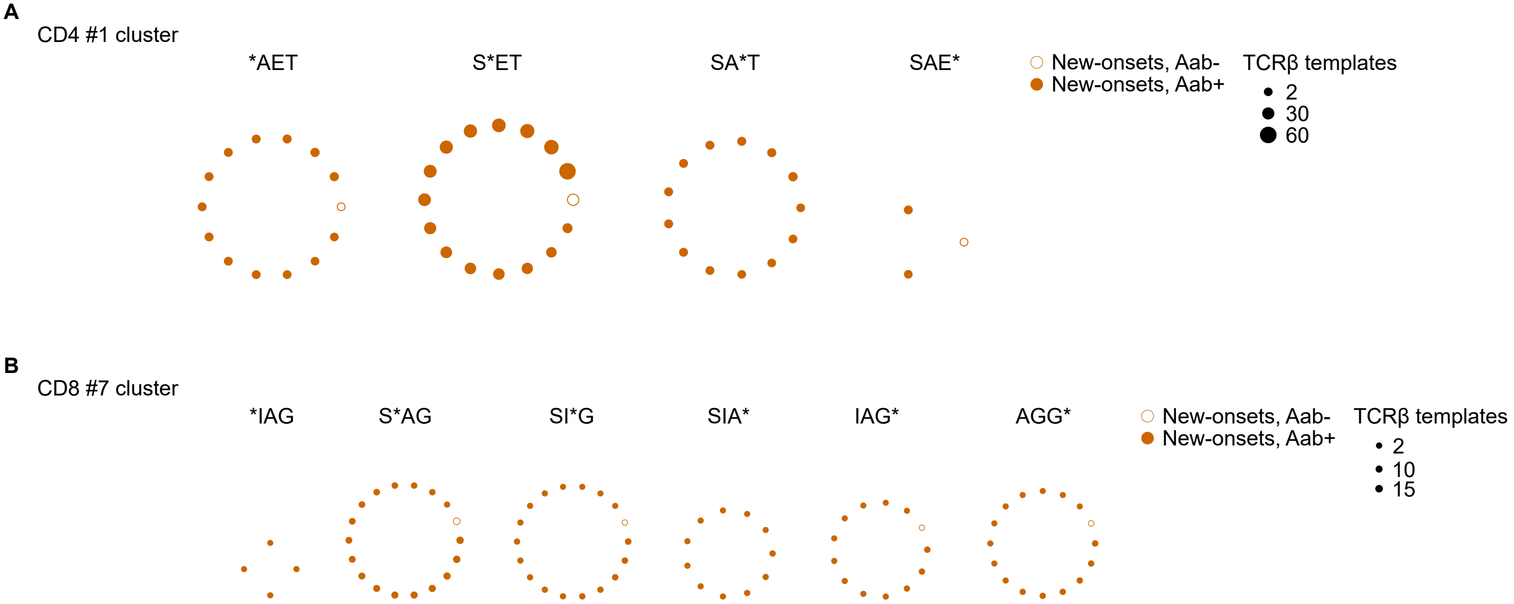

Supplemental Figure 10. Prevalence of clusters containing a preproinsulin-reactive T cell receptor beta chain in a separate cohort of new-onset type 1 diabetes patients. Multivariable plots showing the presence of the (**A**) four clusters for CD4 TCR #1 and (**B**) six clusters for CD8 TCR #7 at the individual level in new-onset T1D patients. Size indicates the TCRβ template number, open circles are autoantibody negative subjects, and closed circles are autoantibody positive subjects.

Table S1. Influenza-reactive T cell receptor beta chain sequences.

TCR ID	T cell Subset	TCRβ Chain			HLA Restriction*	PPI Epitope	TCRβ Total Templates (No.)†	
		V-gene	J-gene	CDR3 Amino Acid Sequence			DAISY Controls (n=25)	DAISY Cases (n=29)
**1**	CD4	09-01	01-02	CASSRGNYGYTF	DR4	Hemagglutinin	2	1
**2**	CD4	28-01	01-02	CASSSTGLPYGYTF	DQ8	Hemagglutinin	0	0
**3**	CD4	06-03	01-05	CASVGGRLDQPQHF	DQ2	Hemagglutinin	0	0
**4**	CD8	19-01	02-07	CASSIRSSYEQYF	A02	Matrix protein	227	133
**5**	CD8	19-01	01-02	CASSIGLYGYTF	A02	Matrix protein	16	10
**6**	CD8	19-01	02-07	CASSIRSGKEQYF	A02	Matrix protein	0	0

*DR4 is the DRB1*04:01 allele

*DQ8 consists of the alleles DQA1*03:01-DQB1*03:02

*DQ2 consists of the alleles DQA1*05:01-DQB1*02:01

*A02 is the A*02:01 allele

†DAISY cases and controls had TCRβ sequencing performed at 4 separate time points

Table S2. Statistics for temporal changes in TCR #1 clusters (CD4 PPI-reactive)

Cluster	Group(s)	Metric	Time Point(s)	p-value**
*AET	Controls	Templates	1 → 2	0.429
			1 → 3	0.083
			1 → 4	0.281
		Diversity	1 → 2	0.608
			1 → 3	0.192
			1 → 4	0.662
	Cases	Templates	1 → 2	0.997
			1 → 3	0.922
			1 → 4	0.830
		Diversity	1 → 2	0.250
			1 → 3	0.259
			1 → 4	0.058
	Controls vs. Cases	Templates	1	0.462
			2	0.320
			3	0.357
			4	0.198
		Diversity	1	0.455
			2	0.859
			3	0.230
			4	0.641
S*ET	Controls	Templates	1 → 2	0.024
			1 → 3	0.010
			1 → 4	<0.001
		Diversity	1 → 2	0.045
			1 → 3	<0.001
			1 → 4	<0.001
	Cases	Templates	1 → 2	0.654
			1 → 3	0.324
			1 → 4	0.972
		Diversity	1 → 2	0.413
			1 → 3	0.004
			1 → 4	0.109
	Controls vs. Cases	Templates	1	0.949
			2	0.582
			3	0.866
			4	0.022
		Diversity	1	0.825
			2	0.523
			3	0.993
			4	0.036

Table S2, cont'd.

SA*T	Controls	Templates	1 → 2	0.598
			1 → 3	0.156
			1 → 4	0.137
		Diversity	1 → 2	0.554
			1 → 3	0.216
			1 → 4	0.074
	Cases	Templates	1 → 2	0.100
			1 → 3	0.100
			1 → 4	0.678
		Diversity	1 → 2	0.615
			1 → 3	0.100
			1 → 4	0.998
	Controls vs. Cases	Templates	1	0.845
			2	0.992
			3	0.990
			4	0.124
		Diversity	1	0.031
			2	0.823
			3	1.000
			4	0.438
SAE*	Controls	Templates	1 → 2	NaN
			1 → 3	NaN
			1 → 4	NaN
		Diversity	1 → 2	NaN
			1 → 3	NaN
			1 → 4	NaN
	Cases	Templates	1 → 2	NaN
			1 → 3	NaN
			1 → 4	NaN
		Diversity	1 → 2	NaN
			1 → 3	NaN
			1 → 4	NaN
	Controls vs. Cases	Templates	1	NaN
			2	NaN
			3	NaN
			4	NaN
		Diversity	1	NaN
			2	NaN
			3	NaN
			4	NaN

**Values shaded in light orange trend toward significance: $p<0.1$; values shaded in red are significant: $p<0.05$. NaN indicates when p-values could not be calculated because sample size was too small. Mixed-effects models were used to compare groups and time points while accounting for the correlation of multiple measures within a participant.

Table S3. Statistics for temporal changes in TCR #2 clusters (CD4 PPI-reactive)

Cluster	Group(s)	Metric	Time Point(s)	p-value**
*LQS	Controls	Templates	1 → 2	NaN
			1 → 3	NaN
			1 → 4	NaN
		Diversity	1 → 2	NaN
			1 → 3	NaN
			1 → 4	NaN
	Cases	Templates	1 → 2	NaN
			1 → 3	NaN
			1 → 4	NaN
		Diversity	1 → 2	NaN
			1 → 3	NaN
			1 → 4	NaN
	Controls vs. Cases	Templates	1	NaN
			2	NaN
			3	NaN
			4	NaN
		Diversity	1	NaN
			2	NaN
			3	NaN
			4	NaN
S*QS	Controls	Templates	1 → 2	0.337
			1 → 3	0.879
			1 → 4	0.959
		Diversity	1 → 2	0.507
			1 → 3	0.977
			1 → 4	0.100
	Cases	Templates	1 → 2	0.951
			1 → 3	0.989
			1 → 4	0.423
		Diversity	1 → 2	0.869
			1 → 3	0.945
			1 → 4	0.244
	Controls vs. Cases	Templates	1	0.072
			2	0.819
			3	0.083
			4	0.709
		Diversity	1	0.009
			2	0.609
			3	0.054
			4	0.751

Table S3, cont'd.

SL*S	Controls	Templates	1 → 2	0.010
			1 → 3	0.007
			1 → 4	0.034
		Diversity	1 → 2	0.058
			1 → 3	0.054
			1 → 4	0.242
	Cases	Templates	1 → 2	0.082
			1 → 3	0.030
			1 → 4	0.007
		Diversity	1 → 2	0.614
			1 → 3	0.132
			1 → 4	0.009
	Controls vs. Cases	Templates	1	0.400
			2	0.883
			3	0.925
			4	0.301
		Diversity	1	0.346
			2	0.495
			3	0.984
			4	0.105
SLQ*	Controls	Templates	1 → 2	NaN
			1 → 3	NaN
			1 → 4	NaN
		Diversity	1 → 2	NaN
			1 → 3	NaN
			1 → 4	NaN
	Cases	Templates	1 → 2	0.730
			1 → 3	0.974
			1 → 4	0.931
		Diversity	1 → 2	0.999
			1 → 3	0.670
			1 → 4	0.858
	Controls vs. Cases	Templates	1	1.000
			2	0.602
			3	0.189
			4	0.558
		Diversity	1	0.472
			2	0.442
			3	0.108
			4	0.722

**Values shaded in light orange trend toward significance: $p<0.1$; values shaded in red are significant: $p<0.05$. NaN indicates when p-values could not be calculated because sample size was too small. Mixed-effects models were used to compare groups and time points while accounting for the correlation of multiple measures within a participant.

Table S4. Statistics for temporal changes in TCR #7 clusters (CD8 PPI-reactive)

Cluster	Group(s)	Metric	Time Point(s)	p-value**
*IAG	Controls	Templates	1 → 2	0.694
			1 → 3	0.883
			1 → 4	0.486
		Diversity	1 → 2	0.989
			1 → 3	0.974
			1 → 4	0.702
	Cases	Templates	1 → 2	NaN
			1 → 3	NaN
			1 → 4	NaN
		Diversity	1 → 2	NaN
			1 → 3	NaN
			1 → 4	NaN
	Controls vs. Cases	Templates	1	0.091
			2	0.986
			3	0.993
			4	0.974
		Diversity	1	0.968
			2	0.367
			3	0.583
			4	0.718
S*AG	Controls	Templates	1 → 2	0.239
			1 → 3	0.035
			1 → 4	0.100
		Diversity	1 → 2	0.267
			1 → 3	0.044
			1 → 4	0.151
	Cases	Templates	1 → 2	0.666
			1 → 3	0.988
			1 → 4	0.595
		Diversity	1 → 2	0.596
			1 → 3	0.986
			1 → 4	0.500
	Controls vs. Cases	Templates	1	0.643
			2	0.944
			3	0.137
			4	0.680
		Diversity	1	0.630
			2	0.954
			3	0.033
			4	0.893

Table S4, cont'd.

SI*G	Controls	Templates	1 → 2	0.938
			1 → 3	0.513
			1 → 4	0.618
		Diversity	1 → 2	0.995
			1 → 3	0.600
			1 → 4	0.761
	Cases	Templates	1 → 2	0.729
			1 → 3	0.993
			1 → 4	0.100
		Diversity	1 → 2	0.977
			1 → 3	0.100
			1 → 4	0.100
	Controls vs. Cases	Templates	1	0.829
			2	0.527
			3	0.405
			4	0.488
		Diversity	1	0.924
			2	0.828
			3	0.290
			4	0.494
SIA*	Controls	Templates	1 → 2	NaN
			1 → 3	NaN
			1 → 4	NaN
		Diversity	1 → 2	NaN
			1 → 3	NaN
			1 → 4	NaN
	Cases	Templates	1 → 2	NaN
			1 → 3	NaN
			1 → 4	NaN
		Diversity	1 → 2	NaN
			1 → 3	NaN
			1 → 4	NaN
	Controls vs. Cases	Templates	1	NaN
			2	NaN
			3	NaN
			4	NaN
		Diversity	1	NaN
			2	NaN
			3	NaN
			4	NaN

Table S4, cont'd.

IAG*	Controls	Templates	1 → 2	0.694
			1 → 3	0.883
			1 → 4	0.486
		Diversity	1 → 2	0.989
			1 → 3	0.974
			1 → 4	0.702
	Cases	Templates	1 → 2	NaN
			1 → 3	NaN
			1 → 4	NaN
		Diversity	1 → 2	NaN
			1 → 3	NaN
			1 → 4	NaN
	Controls vs. Cases	Templates	1	0.091
			2	0.986
			3	0.993
			4	0.974
		Diversity	1	0.968
			2	0.367
			3	0.583
			4	0.718
AGG*	Controls	Templates	1 → 2	0.100
			1 → 3	0.46
			1 → 4	0.998
		Diversity	1 → 2	0.818
			1 → 3	0.985
			1 → 4	0.972
	Cases	Templates	1 → 2	0.644
			1 → 3	0.309
			1 → 4	0.352
		Diversity	1 → 2	0.847
			1 → 3	0.797
			1 → 4	0.333
	Controls vs. Cases	Templates	1	0.274
			2	0.933
			3	0.946
			4	0.490
		Diversity	1	0.219
			2	0.751
			3	0.684
			4	0.399

**Values shaded in light orange trend toward significance: $p<0.1$; values shaded in red are significant: $p<0.05$. NaN indicates when p-values could not be calculated because sample size was too small. Mixed-effects models were used to compare groups and time points while accounting for the correlation of multiple measures within a participant.

Table S5. Demographic and immunologic characteristics of new-onset T1D subjects.

	**New-onset T1D Cohort (n=143)**
**Age,** mean (SD) in years	12.1 (4.2)
**Gender**	
Female % (number)	45% (n=65)
**Race/Ethnicity**	
Non-Hispanic	85% (n=122)
Hispanic	14% (n=20)
Not reported	1% (n=1)
**T1D duration,** days	
Mean (SD)	4.9 (9.1)
Median	2.0
Range	0.0 – 55.0
**T1D Autoantibodies,** % (number)	
Present	94% (n=135)
Absent	5% (n=7)
Not measured	1% (n=1)
**TCRβ Templates,** number	
Mean	158,236
Median	150,194
Range	10,153 – 381,381
**HLA-DQ Genotype,** % (number)	
DQ8/X*	40% (n=57)
DQ2/X**	25% (n=36)
DQ2/8	26% (n=37)
DQX/X	9% (n=13)

SD = standard deviation; T1D = type 1 diabetes
*DQ8 consists of the alleles DQA1*03:01-DQB1*03:02 and DQA1*03:03-DQB1*03:02
**DQ2 consists of the subtypes: DQA1*05:01-DQB1*02:01, DQA1*02:01-DQB1*02:02, DQA1*03:01-DQB1*02:02, or DQA1*03:02-DQB1*02:02
